# Frequency dependent growth of bacteria in living materials

**DOI:** 10.3389/fbioe.2022.948483

**Published:** 2022-09-08

**Authors:** Daniel D. Lewis, Ting Gong, Yuanwei Xu, Cheemeng Tan

**Affiliations:** ^1^ Department of Biomedical Engineering, University of California, Davis, CA, United States; ^2^ Integrative Genetics and Genomics, University of California, Davis, CA, United States; ^3^ Department of Biomedical Engineering, Peking University, Beijing, China

**Keywords:** bacteria, synthetic biology, nonlinear dynamics, growth, materials, engineered living materials

## Abstract

The fusion of living bacteria and man-made materials represents a new frontier in medical and biosynthetic technology. However, the principles of bacterial signal processing inside synthetic materials with three-dimensional and fluctuating environments remain elusive. Here, we study bacterial growth in a three-dimensional hydrogel. We find that bacteria expressing an antibiotic resistance module can take advantage of ambient kinetic disturbances to improve growth while encapsulated. We show that these changes in bacterial growth are specific to disturbance frequency and hydrogel density. This remarkable specificity demonstrates that periodic disturbance frequency is a new input that engineers may leverage to control bacterial growth in synthetic materials. This research provides a systematic framework for understanding and controlling bacterial information processing in three-dimensional living materials.

## Introduction

The integration of designer microbes with synthetic materials has birthed a new class of customizable, environmentally-responsive living materials (Cao et al., 2017; Hay et al., 2018; Balasubramanian et al., 2019; Gonzalez et al., 2020; Gilbert et al., 2021; Manjula-Basavanna et al., 2021). Genetic circuits inside bacteria can be designed to produce enzymatic or structural components based on intra- or extra-cellular cues. Through expression regulation mediated by genetic circuits, designer microbes can control self-assembly (Cao et al., 2017) and adaptation (Hay et al., 2018; Gonzalez et al., 2020; Gilbert et al., 2021) in synthetic materials. Designer microbes are often incorporated into hydrogels to form engineered living materials (Chyzy et al., 2020). Hydrogels’ physical properties, such as stiffness, density, and viscosity, play an important role in the metabolic activity and growth rate of encapsulated cells (Gilbert et al., 2021; Manjula-Basavanna et al., 2021). While the interactions between hydrogels and cellular growth can be exploited to control self-assembly (Cao et al., 2017), they can also impart metabolic heterogeneity. Metabolic heterogeneity of encapsulated cells in synthetic materials is known to impede their growth and genetic circuit activity (Wessel et al., 2013 Pabst et al., 2016; Alvarez et al., 2020). To expand the utility and range of biological functions in engineered living materials, there is a critical need to understand and control the growth of synthetically encapsulated microbes.

Foundational work on natural bacterial mechanotransduction has shown that cells are capable of integrating complex environmental inputs into decisions that reshape cellular physiology (Taute et al., 2015; Dufrene et al., 2020). However, these studies have also revealed gaps in our understanding of how bacteria integrate temporally and spatially heterogeneous signals into beneficial phenotypic changes (Kandemir et al., 2018; Sanfilippo et al., 2019). There have been explorations of the connections between spatial structure and information processing in microbial colonies (Kong et al., 2017; van Vliet et al., 2018; Alnahhas et al., 2019; Bittihn et al., 2020; Dal Co et al., 2020; Gupta et al., 2020; Kussell and Leibler, 2021; Zhang et al., 2021), but these studies have not addressed the information processing of microbes embedded in three-dimensional synthetic materials. Along this line, studies using engineered cells suggest that phenomena like Turing patterns may facilitate collective decision-making based on spatial and temporal information (Cao et al., 2020; Karig et al., 2020). Do unique information-processing principles govern microbial behavior in three-dimensional materials? If so, could those principles be used to control the growth of microbes encapsulated in synthetic materials?

Here, we study the capacity of bacteria to capitalize on rapid periodic disturbances in a synthetic hydrogel. Rapid periodic disturbances are common in complex 3D environments that are key targets for engineered living materials (Nguyen et al., 2018), such as cardiac tissue, airways, and intestines. Modulating growth of synthetic bacteria in these environments is critical for maintaining homeostasis with the host microbiome and retaining biological efficacy of engineered living materials. We use an antibiotic resistance gene to explore how the metabolic trade-off of a synthetic genetic module affects bacterial growth in an encapsulated and temporally fluctuating environment. This work uncovers a counterintuitive phenomenon where the frequency of rapid periodic disturbances optimizes the growth of antibiotic-resistant bacteria encapsulated in hydrogel. A conceptual model recapitulates this frequency-dependent optimization of growth by proposing that cells can leverage stochastic resonance (Russell et al., 1999; Paulsson et al., 2000; Bahar and Moss, 2004; McDonnell and Abbott, 2009; Zeng et al., 2009; Wiesenfeld and Moss, 2018) to integrate spatial and temporal information to survive in synthetic materials.

## Results

### Bacteria exhibit fluctuating ATP metabolism in periodically perturbed hydrogel

**FIGURE 1:**
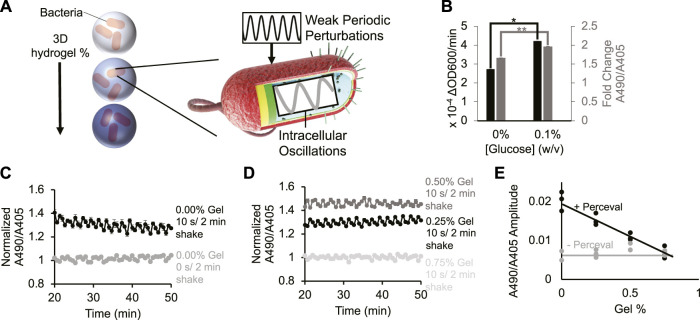
Periodic environment perturbations can propagate to intrabacterial metabolite oscillations despite hydrogel encapsulation. **(A)** Cartoon depicting bacteria encapsulated in varying densities of hydrogel being subjected to external kinetic perturbation. Experiment assays whether external perturbations propagate to internal fluctuations in cellular metabolites. **(B)** Bacterial growth rate (black bar) and their Perceval expression (grey bar) in M9 minimal medium (0% and 0.1% glucose, respectively). Difference between bacterial growth rates is significant (*p*-value < 0.05, indicated by asterisk). Difference between Perceval values is significant (*p*-value < 0.005, indicated by two asterisks). Bar height represents mean values, and error bars represent standard error of the mean. Mean and SEM are calculated using four biological replicates. **(C)** Intrabacterial ATP/ADP ratio over time with 0 or 10 s/2 min shaking. Absorbance at 490 nm to absorbance at 405 nm ratio represents ATP/ADP ratio. Basal fluctuation in A490/A405 without shaking is likely caused by the mechanical movement of the platereader to read the samples. Points represent mean values, and error bars represent standard error of the mean. Mean and SEM are calculated using four biological replicates. **(D)** Intrabacterial ATP/ADP ratio at different hydrogel densities (0.25%–0.75% gel). Absorbance at 490 nm to absorbance at 405 nm ratio suggests that periodic perturbation of hydrogel encapsulated cells can propagate to changes in intracellular metabolites. Points represent mean values, and error bars represent standard error of the mean. Mean and SEM are calculated using four biological replicates. **(E)** Amplitude of ATP/ADP fluctuations at 0.5 cycles/min in *Escherichia coli* DH5α cells (grey line) and Perceval cells (black line) shaken for 10 s/2 min at different gel densities (0%–0.75%). Results show that increasing hydrogel density reduces amplitude of ATP/ADP ratio fluctuation. Points represent individual replicates, line highlights trend. At least two replicates per condition.

Hydrogel encapsulation is known to disrupt cellular metabolism underlying signal propagation (Shao et al., 2017). However, it was unknown whether these disruptions in metabolism could prevent a cell from responding to rapid fluctuations in its environment. To address this unknown, we grew bacteria in a culture system where we could modulate the gel density of the media, periodically perturb the cells, and measure the subsequent changes in intracellular signals (Figure 1A). *E. coli* DH5*α* was used as a basal strain, cultured in freshly-prepared LB (Luria Broth) media and hydrogel. Encapsulation conditions were controlled by dissolving hydrogel in media during autoclaving, then storing the media at 37°C until use (Methods- Section A).

To monitor changes in intracellular metabolites, bacteria were grown expressing the metabolic reporter known as Perceval (Berg et al., 2009) (Methods- Section B and C). Perceval is designed to fluorescently report the ratio of ATP to ADP. Increased bacterial growth rate (Figure 1B, black bar, *p*-value: 0.02) correlated with an increased ratio of absorbance at 490 nm to absorbance at 405 nm (Figure 1B, grey bar, *p*-value: 0.001). Since the ratio of A490/A405 corresponds to the ATP/ADP ratio, these results suggest that the Perceval reporter can serve as a proxy for cellular bioactivity. These results are consistent with the previous use of ATP as a reporter of metabolic activity (Lopatkin et al., 2019).

Next, bacteria expressing Perceval were cultured in a periodically-shaken environment and changes in the ATP to ADP ratio were measured over time at different gel densities. Cells were shaken at 0 s/2 min (i.e., 0 s every 2 min) or 10 s/2 min (i.e., 10 s every 2 min), and absorbance at 490 nm and 405 nm was measured three times in that 2-min interval. In liquid cultures shaken for 10 s/2 min, oscillations occurred in the A490/A405 ratio (Figure 1C black). Some A490/A405 fluctuation was observed in the absence of shaking (Figure 1C grey), but this is likely due to the mechanical movement required to measure different wells every 2 minutes. When hydrogel-encapsulated bacteria were shaken for 10 s/2 min, oscillation in the A490/A405 ratio was also recorded (Figure 1D black, dark grey). These results challenge the assumption that hydrogel encapsulation eliminates the dynamic responsiveness of intracellular metabolites to environmental conditions. High gel densities were able to repress oscillations in the A490/A405 ratio (Figure 1D light grey). These results suggest that sufficiently concentrated environmental hydrogel can dampen short-term cellular metabolic responses. A power spectral analysis (Figure 1E; Supplementary Figure S1 2nd Column) revealed that Perceval-expressing cells shaken for 10 s/2 min at 0.25% gel experienced significantly a higher oscillation amplitude at 0.5 cycles/min (*p*-value: 0.002) than cells without Perceval shaken at the same frequency. We observed similar periodicity in bacteria expressing a luminescent reporter under periodic and hydrogel-encapsulating conditions Supplementary Figure S2, Methods- Section D). These results demonstrate that rapidly oscillating physical perturbation of hydrogel-encapsulated bacteria can propagate to intracellular metabolic changes (Figures 1C–E; Supplementary Figures S1, S2).

### Bacterial gene expression and growth rate can be controlled by exogenous hydrogel

**FIGURE 2:**
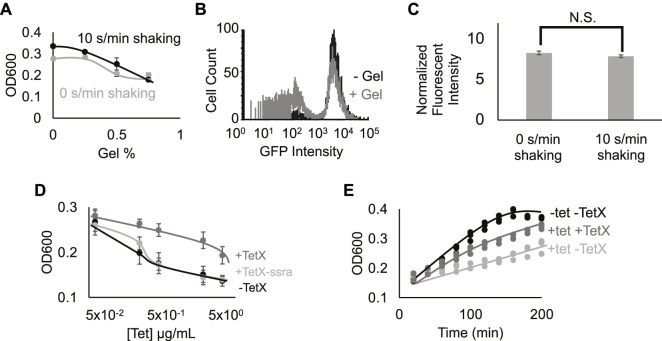
Hydrogel encapsulation imparts gene expression heterogeneity. **(A)** Cell density in different gel density environments at 2 h 0%, 0.25%, 0.5%, and 0.75% gel density with 10 s/min shaking (black line) or with 0 s/min shaking (grey line). Increased hydrogel density reduces bacterial growth. Points represent mean values, and error bars represent standard error of the mean. Mean and SEM are calculated using six biological replicates. **(B)** Fluorescence intensity distribution of cells expressing TetX-GFP fusion protein in media containing 0% (black line) and 0.5% low-melting-temperature agarose (grey line). Results demonstrate that hydrogel-encapsulation limits the expression of TetX-GFP in a subset of bacteria. One of two replicates shown for each condition, refer to Supplementary.Figure S4 for both replicates. **(C)** Fluorescence intensity of TetX-GFP normalized by OD600 of cells after 2 h of growth with either 0 s/min or 10 s/min shaking. Mean expression levels are not significantly different. Two-tailed, equal variance *t*-test returns *p*-value = 0.189. N.S. stands for “not significant”. Bar height represents mean values, and error bars represent standard error of the mean. Mean and SEM are calculated using eight biological replicates. **(D)** Cell density of resistant and non-resistant DH5*α* cells under different concentrations of tetracycline. +TetX-ssra (light grey line), +TetX (grey line) and -TetX (black line). Results show that TetX rescues bacterial growth from tetracycline repression. Points represent mean values, and error bars represent standard error of the mean. Mean and SEM are calculated using six biological replicates. **(E)** Cell density of resistant bacteria (TetX) in different tetracycline (tet) over time. 0.625 µg/ml tetracycline on DH5*α* cells without TetX (light grey line), 0.625 µg/ml tetracycline on DH5*α* cells without TetX (grey line) and 0 µg/ml tetracycline on DH5*α* cells without TetX (black line). Results show that tetracycline inhibition of cellular growth is reflected by decreased rates of growth over time, and rescue by TetX is reflected by increased rates of growth over time. Points represent individual replicates. Six replicates per condition at each time point.

To confirm that the hydrogel used in this study imparts heterogeneity on growing cells, bacterial growth in a 3D hydrogel was measured. Bacterial density was recorded for 2 h under growth conditions with a range of hydrogel densities (Supplementary Figures 3A–D top rows). Bacterial density at 2 h was used to compare growth between cultures, serving as a surrogate reporter of cellular bioactivity (Figure 2A). The 2-h time point was chosen because the rate of change in OD600 decreased after 2 h. The results showed that increasing gel density from 0% to 0.75% (weight/volume) caused a 1.8-fold reduction in final bacterial density in cells shaken for 10 s/min (Figure 2A). This suggests that DH5*α* cells grew slower with increasingly dense hydrogel.

To establish the effect of exogenous hydrogel on gene expression modules, DH5*α* cells constitutively expressing GFP (Methods- Section B) were grown in media with 0.5% gel (w/v) or 0% gel. Single-cell expression data from DH5*α* cells grown in the presence of hydrogel showed that a secondary population of low-fluorescence cells appeared (Figure 2B; Supplementary Figure S4, Methods- Section E). This bimodal distribution suggests that hydrogel encapsulation imparts heterogeneous growth conditions that stochastically limit expression levels from the gene module, splitting the cellular population into “on” and “off” states. Taken together, hydrogel-mediated reductions in growth and gene expression are consistent with previous work on microbes in heterogeneous environments (Alvarez et al., 2020; Pabst et al., 2020; Wessel et al., 2020). Notably, changes in periodic perturbation did not significantly alter fluorescence measurements (Figure 2C; Supplementary Figure S5). Prior work has shown that faster bacterial growth can lead to a faster protein dilution rate, balancing the increase of protein synthesis rate, leading to balanced protein level (Klumpp and Hwa, 2014). This phenomenon may explain why periodic perturbations did not lead to a boost in protein expression from the constitutive genetic module.

### Engineered genetic module imparts functionality on encapsulated bacteria

To test the effect of protein expression on bacterial growth during encapsulation, a genetic module that constitutively expresses TetX was constructed (Ghosh et al., 2015) (Methods- Section A). We validated the efficacy of this TetX module by growing resistant and non-resistant bacteria at a series of tetracycline concentrations, demonstrating that TetX expression allows cells to grow to a 1.5-fold greater final density in the presence of 5 μg/ml tetracycline (Figure 2D). This change in density is also reflected over time (Figure 2E). To confirm that the protein product of the module (i.e., TetX) was imparting antibiotic resistance, an unstable version of TetX was constructed by fusing a degradation tag to TetX (Gottesman et al., 1998). The unstable protein either imparted very weak tetracycline resistance or no detectable tetracycline resistance (Figure 2D). These results establish that TetX expression, rather than nonspecific protection from the TetX module, allows cells to effectively resist tetracycline (Figures 2D,E).

### Bacteria exhibit emergent growth dynamics in a 3D fluctuating hydrogel

**FIGURE 3:**
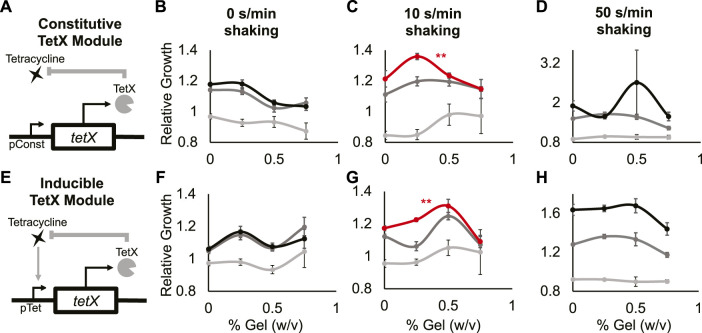
A minimal antibiotic-resistance module enables frequency-dependent response of bacterial growth in hydrogel. **(A)** Abstract depiction of tetracycline degradation by constitutive TetX expression module. **(B–D)** Relative growth of *E. coli* cells constitutively expressing TetX with 0 s/min shaking **(B)**, with 10 s/min shaking **(C)**, with 50 s/min shaking **(D)**. Light grey is 0 µg/ml tetracycline, dark grey is 0.3125 µg/ml tetracycline, black is 0.625 µg/ml tetracycline. The red line represents a statistically significant biphasic curve as judged by a Bonferroni multiple comparisons test (cells treated with 0.625 µg/ml tetracycline). Points represent mean values, and error bars represent standard error of the mean. **(B)** and **(C)** Mean and SEM are calculated using six biological replicates. **(D)** Mean and SEM are calculated using four biological replicates. **(E) **Abstract depiction of feedback between bacterial growth and inducible TetX expression module when feeding tetracycline. **(F–H)** Relative growth of *E. coli* cells inducibly expressing TetX with 0 s/min shaking **(F)**, with 10 s/min shaking **(G)**, with 50 s/min shaking **(H)**. Light grey is 0 µg/ml tetracycline, dark grey is 0.3125 µg/ml tetracycline, black is 0.625 µg/ml tetracycline. The red line represents a statistically significant biphasic curve as judged by a Bonferroni multiple comparisons test (cells treated with 0.625 µg/ml tetracycline). Points represent mean values, and error bars represent standard error of the mean. Mean and SEM are calculated using six biological replicates.

To examine the population-level consequences of functional protein expression for bacteria inside a periodically-perturbed hydrogel, the growth of DH5*α* cells constitutively expressing TetX was measured over time (Figure 3A, Methods- Section F). The media was supplemented with 0, 0.3125, or 0.625 µg/ml tetracycline. To quantify relative growth, the final density of cells expressing the TetX module was normalized by initial density and the growth of naïve cells cultured under the same conditions. Under all growth conditions, larger concentrations of tetracycline increased the relative growth of TetX-expressing cells. The significance of increases or decreases in relative growth between cells at each pairwise combination of hydrogel densities was assayed using a Bonferroni multiple comparisons test (Methods- Section G). This statistical test was used to classify trends as increasing, biphasic, decreasing, or constant (Supplementary Figure S6).

At 0 s/min shaking and 0.625 or 0.3125 µg/ml tetracycline, the relative growth of cells with the TetX module decreased as gel density increased (Figure 3B black, dark gray; Supplementary Figure S6A 2nd and 3rd columns, Supplementary Figure S7A mid and bottom rows). With 0 s/min shaking and 0 µg/ml tetracycline, relative growth did not significantly change as gel density increased (Figure 3B light gray; Supplementary Figure S6A 1st column, Supplementary Figure S7A top row). At 10 s/min shaking and 0.625 µg/ml tetracycline, cells with the TetX module exhibited a significant biphasic change in relative growth as gel density increased (Figure 3C red; Supplementary Figure S6B 3rd column, Supplementary Figure S7B bottom row). With 10 s/min shaking and 0 or 0.3125 µg/ml tetracycline, the change in relative growth was visually biphasic, but had no significant trend (Figure 3C, dark and light grey; Supplementary Figure S6B 1st and 2nd columns, Supplementary Figure S7B top and mid row). These results suggest that periodic perturbation interacts with TetX expression to allow bacteria to enhance growth in a manner sensitive to hydrogel density.

To distinguish whether biphasic growth was a general response to the presence of shaking or a unique response to a specific frequency of shaking, we gathered additional data from cells shaken at 50 s/min. At 50 s/min shaking and 0.3125 µg/ml tetracycline, relative growth decreased as hydrogel density increased (Figure 3D, dark grey; Supplementary Figure S6C 2nd column, Supplementary Figure S7C mid row). At 50 s/min shaking and 0 or 0.625 µg/ml tetracycline, relative cell growth did not significantly change (Figure 3D black and light grey; Supplementary Figure S6C 1st and 3rd columns, Supplementary Figure S7C top and bottom rows). These results suggest the biphasic response of TetX-expressing cells grown with 10 s/min shaking and 0.625 µg/ml tetracycline (Figure 3C red) is based on the frequency of periodic perturbation rather than the presence of perturbation.

To test whether the frequency-specific and biphasic relative growth curve was mediated by the expression dynamics of the constitutive TetX module, we built and tested an inducible TetX module under the same shaking conditions and hydrogel densities. Inducible TetX expression was achieved by transforming the constitutive TetX module into a cell line (DH5*α*pro) that regulated module expression *via* genomically integrated *tetR* (Figure 3E; Supplementary Figure S8, Methods- Section B). At 0 s/min shaking, no tetracycline concentrations produced a significant change in relative growth as hydrogel density rose (Figure 3F; Supplementary Figures S6D, 9). At 10 s/min shaking and 0.625 µg/ml tetracycline, the inducible TetX module displayed a significant biphasic relationship between relative growth and hydrogel density (Figure 3G red; Supplementary.Figure S6E 3rd column, Supplementary.Figure S9B bottom row). This biphasic growth curve of the inducible module was shifted to the right of the constitutive module growth curve, suggesting that the activation kinetics of TetX production affect population-level integration of spatial and temporal information. At 10 s/min shaking and 0 or 0.3125 µg/ml tetracycline, bacteria inducibly expressing TetX experienced no significant change in relative growth as hydrogel density rose (Figure 3G light and dark grey; Supplementary Figure S9B top and middle rows). At 50 s/min shaking and 0, 0.3125, or 0.625 µg/ml tetracycline, the inducible TetX module similarly displayed no significant change in relative growth as hydrogel density increased (Figure 3H; Supplementary Figures S6D, S9C). Taken together, the results from the constitutive and inducible TetX modules (Figure 3; Supplementary Figures S6, S9) demonstrate that the genetic regulation of an antibiotic resistance gene tunes the hydrogel sensitivity of bacterial growth under tetracycline exposure.

### A conceptual model can explain the emergent growth dynamics of bacteria

**FIGURE 4:**
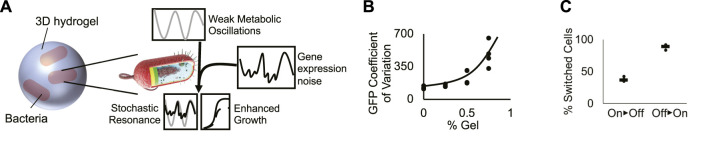
A conceptual model generates frequency-dependent bacterial growth. **(A)** Cartoon of the proposed model of bacterial stochastic resonance. At a specific shaking frequency, weak metabolic oscillations resonate with stochastic fluctuations in TetX production, boosting growth in tetracycline-treated bacteria. **(B)** Coefficient of variation of bacterial GFP expression in hydrogel. Graph shows that increased hydrogel density increases the variability of genetic module expression. Points represent individual replicates, line highlights trend. Four replicates per condition. Line highlights the tend. **(C)** Quantification of “on” and “off” cellular populations that grew from cells sorted in the opposite state. Results suggest that gene expression can switch between “on” and “off” expression modes. Points represent individual replicates. Lines represent the median proportion of switched bacteria. Four replicates per condition.

After observing counterintuitive, frequency-sensitive bacterial growth, we sought to explain this phenomenon. Since expression levels from the genetic module are similar between different shaking frequencies (Figure 2C), differences in mean expression level are unlikely to drive biphasic growth with respect to hydrogel density. Alternatively, frequency-specific, biphasic responses in a noisy environment have been previously associated with stochastic resonance in biological systems (Russell et al., 1999; Paulsson et al., 2000; Bahar and Moss, 2004; McDonnell and Abbott, 2009; Zeng et al., 2009; Wiesenfeld and Moss, 2018). For stochastic resonance theory to apply, there must be a weak oscillatory force and a source of biochemical noise (Figure 4A).

The growth rate characterization experiments for Perceval show that there is an approximately 18% difference between the ATP/ADP ratio associated with cell cultures that exhibit significantly different growth rates (Figure 1B). Maximum versus minimum A490/A405 measurements of cells shaken for 10 s/2 min in liquid media show the peak versus the trough of ATP/ADP oscillations (2x amplitude) have a ∼6% difference in intensity (Figures 1C,D). These results show that shaking can exert an oscillatory force on bacterial metabolism, but that the oscillatory force is not necessarily strong enough to be associated with a significant change in growth rate. This, in turn, suggests that periodic shaking exerts a weak oscillatory influence on growth.

Hydrogels have been hypothesized to generate extrinsic noise in encapsulated bacteria. Because real-time fluctuation of protein levels in single planktonic cells under shaking conditions cannot be measured, we used flow cytometry to infer the effect of hydrogel encapsulation on gene expression noise. Bacteria encapsulated in hydrogel experience an increase in the coefficient of variation of fluorescent reporter expression (Figure 4B; Supplementary Figure S10) caused by the splitting of the population between “on” and “off” expression modes (Figure 2B). We tested the ability of cells grown in hydrogel to switch between “on” and “off” states by sorting the two populations, growing them independently, then quantifying changes in the proportion of “on” vs. “off” cells (Methods- Section H). A mixture of “on” and “off” states were observed in cultures grown from cells started from exclusively one state. These results suggest that cells dynamically switch between “on” and “off” states. These results build on prior work showing that synthetic materials can increase bacterial heterogeneity (Alvarez et al., 2020; Pabst et al., 2020; Wessel et al., 2020), demonstrating that cells grown in hydrogel exhibit dynamic fluctuations in gene expression (Figure 4C). These experiments suggest that hydrogel-mediated dynamic fluctuations in TetX expression impart noise on the growth rate of encapsulated cells.

## Discussion

Encapsulation of designer bacteria into hydrogels has birthed a new class of smart devices that can internally coordinate decision-making. These engineered living materials require novel strategies to maintain the potency of their biological components under restrictive metabolic conditions. To address this need, the effects of internal genetic machinery and external kinetic perturbation were systematically investigated in bacteria encapsulated in a three-dimensional hydrogel. An antibiotic resistance module was shown to optimize bacterial growth under restrictive encapsulation conditions based on the frequency of a rapid periodic disturbance.

Based on the observation of cells using heterogeneity to optimize growth under specific perturbation frequencies, a conceptual model of noise-exploitation was constructed (Supplementary Material, Methods- Section I). This conceptual model postulated that when the periodic growth boost from intermittent shaking resonated with gel-induced fluctuations in TetX expression, simulated cell growth is enhanced, causing a biphasic relationship between growth and gel density (Supplementary Figure S11). These results suggest that stochastic resonance may explain the frequency-specific biphasic growth trends observed in the experimental data (Figures 3C,G, red lines). However, turning the conceptual model into a mechanistic model will require additional quantitative information for parameter and model fitting, such as real-time tracking and measurement of single-cell gene expression in a semisolid environment. Our results do not currently rule out other theories for cellular noise exploitation in fluctuating environments, such as Entrainment of Oscillatory Signals (Gonze et al., 2002), Stochastic Dithering (McDonnell and Abbott, 2009), and Band-pass Filtering (Tan et al., 2007).

To explain why the optimal gel density for relative growth was different between the constitutive and inducible TetX producing cells, the basal growth rates of the two lines were analyzed. The basal growth rate of constitutive TetX-expressing cells was significantly higher than inducible TetX-expressing cells (Supplementary Figure S12 *p*-value: 3 × 10^–5^), likely because cells with the constitutive circuit had higher initial levels of TetX when they were exposed to tetracycline. Further simulations showed that increased basal growth rates (represented by η in Supplementary Equation S1) reduced the optimal gel density for bacterial growth governed by stochastic resonance (Supplementary Figure S12B). These models do not rule out the possibility that other phenomena, such as microcolony-mediated antibiotic protection, could change the optimal gel density for bacterial growth affected by stochastic resonance. However, these models demonstrate that the decreased basal growth rate of inducible TetX-expressing cells is sufficient to explain the shift observed in the biphasic relative growth curve data. These results show how the metabolic trade-off of constitutive versus inducible TetX production changes bacterial integration of spatial and temporal information.

The phenomenon of frequency-dependent growth is an attractive target for the future design of new engineered living materials. Many tissues experience rapid periodic disturbances, incentivizing the construction of genetic modules that are “tuned” to a unique environmental frequency. Frequency-responsive materials could be used to target environments with specific densities for drug delivery or physical reinforcement.

This study invites several lines of future inquiry. New strains and encapsulated substrates may be tested to create more systematic guidelines for the application of frequency-optimized growth to engineered living materials. To explain why constitutive TetX-producing cells have an optimal hydrogel density distinct from inducible TetX-producing cells, additional genetic circuits could be used to explore which regulatory features mediate the integration of spatial and temporal information. Further high-level evidence for noise exploitation could also be collected by identifying a periodic shaking frequency that shifts the biphasic growth curve rather than eliminating it.

## Methods

### Contact for reagent and resource sharing

Further information and requests for resources and reagents should be directed to and will be fulfilled by the Lead Contact, Cheemeng Tan (cmtan@ucdavis.edu).

#### A Media preparation and handling

All media in this study was prepared from granulated Miller’s Luria Broth from Research Products International. Seaplaque low-melting-temperature agarose (VWR) was incorporated into the media before autoclaving. Media was autoclaved for 30 min at 121°C. Sterilization and pressure release took 1 h, after which media was cooled on the bench for 10 min before being placed in a 37°C incubator and being shaken at 200 rpm. During the course of experiments where cells were grown with low-melting-temperature-agarose, media was placed on a hotplate kept at 40°C while it was in use, then returned to the shaker when it was not in use. Media was made fresh the day before every experiment.

Consistent sterilization time and continuous heating of media containing low-melting temperature agarose were found to be critical for the reproducibility of all results. Densities of low-melting-temperature agarose above 0.75% increased the variability of the individual replicates such that reproducible results were not possible and were thus excluded from this study.

#### B Plasmid construction and expression control

All plasmids used in this study were generated by Gibson cloning. The coding sequence for TetX was ordered from Genscript. The TetX sequence, the GFP sequence, all promoters, ribosome binding sites, and degradation tags were amplified by PCR with Q5 polymerase (NEB), gel extracted (Qiagen), and quantified using a Nanodrop (Thermo Fisher). For the constitutive and inducible circuits, TetX and GFP sequences were assembled with P_Tet_, RBS, and ssra tags as necessary in the pZ backbone that had been digested with *Eco*RI and *Bam*HI with buffer 3.1 (all NEB) at 37°C in a static incubator overnight. For the inducible circuits, expression was controlled by a gnomically integrated copy of TetR in the line DH5*α*pro. DNA fragments were assembled *via* the Gibson Assembly Master Mix (NEB) for 1 h at 50°C following the DNA molar ratios suggested by the company’s protocol. Successful transformations were assayed by digestion with *Nco*I, *Bam*HI, and buffer 3.1 and confirmed by Sanger sequencing. The plasmid for the luminescence tests was pCS-P_EsaR_-P_lux_, a gift from Cynthia Collins (Addgene plasmid #47655). *E. coli* DH5α/pRsetB-his7-Perceval was a gift from Gary Yellen’s lab (Addgene plasmid #20336). Plasmid pRsetB-his7-Perceval was isolated and transferred into *E. coli* DH5α/pSC-Ptet-T7 which expresses T7 polymerase constitutively.

#### C Perceval characterization of the effect of periodic shaking on cellular metabolism

A single colony of *E. coli* DH5α/pSC-P_tet_-T7/pRsetB-his7-Perceval was picked and inoculated into LB with 50 μg/ml kanamycin and 100 μg/ml carbenicillin overnight in 3 ml in a 14 ml test tube at 37°C at 120° tilt from horizontal with 200 rpm shaking.

For quantification of ATP/ADP in M9 media, the overnight culture was subcultured into M9 with 0.05%, 0.1%, 0.2%, and 0.4% glucose with the corresponding antibiotic with an initial OD_600 nm_ of 0.05. Next, 200 µl cell culture was put inside 96 well black plate with a clear flat bottom (Corning, ref #3631) combined with a clear lid. ATP/ADP ratios were measured by taking three rounds of reading at excitation 495 nm/emission 530 nm, and 405 nm/emission 530 nm by plate reader in a 2 min cycle with 10 s orbital shaking (amplitude 3) each cycle. *T*-tests were performed in Microsoft Excel using two tails and unequal variance.

For quantification of ATP/ADP in LB medium, the overnight culture was subcultured into fresh LB at 37°C at 120° tilt from horizontal with 200 rpm shaking with initial OD_600 nm_ = 0.05, A490 and A405 values of *E. coli* DH5α/pSC-P_tet_-T7/pRsetB-his7-Perceval in LB medium with glucose were recorded using plate reader. ATP/ADP ratios were measured by taking three rounds of reading at excitation 495 nm/emission 530 nm, and 405 nm/emission 530 nm by plate reader in a 2 min cycle with 10 s orbital shaking (amplitude 3). Subsequent readings were analyzed by Fourier transform in MATLAB using the fft function. Significance between differences in fluctuation amplitude was measured by a two-tailed, unequal variance *t*-test in Microsoft Excel.

#### D Luminescent characterization of the effect of periodic shaking on cellular metabolism

All luminescence results were generated using a plate reader (Tecan M1000pro). Cells were grown overnight at 37°C at 120° tilt from horizontal with 200 rpm shaking in LB with antibiotics from a single colony, then diluted to a constant OD600 value of 0.5. All cell lines were incubated on ice for 30 min. Cell cultures standardized in this way were further diluted 1:100 into LB media with the appropriate antibiotics and the appropriate density of low-melting-temperature agarose as indicated in the data. DH5α cells were grown in media without antibiotics, the luminescent cell line was grown with 30 µg/ml kanamycin. These cells were incubated for 1.5 h at 37°C at 120 tilt from horizontal with 200 rpm shaking until they reached the beginning of exponential growth, at which point they were distributed into a 96-well plate, covered with a lid, and put into the plate reader at 37°C. Total well volume was 200 µl. The plate reader subjected the cells to 10 s of orbital shaking (amplitude 3), iterated through three rounds of reading luminescence then OD600, then restarted the cycle. Each cycle took 2 min. Readings were taken for at least 6 h. Comparisons between shaking and non-shaking conditions were made by picking times points where the OD600 readings of the shaking and non-shaking cultures were equal to 0.25.

#### E Flow cytometry for cells expressing GFP

DH5α cells containing the pZ (P_Const_-GFP) construct were grown overnight at 37°C at 120 tilt from horizontal with 200 rpm shaking in LB with 30 µg/ml kanamycin from a single colony, then diluted to a constant OD600 value of 0.5. Cells were incubated on ice for 30 min, then further diluted 1:100 into LB media with 30 µg/ml kanamycin and the appropriate density of low-melting-temperature agarose as indicated in the data. These cells were incubated for 1.5 h at 37°C at 120° tilt from horizontal with 200 rpm shaking until they reached the beginning of exponential growth, at which point they were distributed into a 96-well plate, covered with a lid, and put into the plate reader at 37°C. Total well volume was 200°µl. OD600 and GFP fluorescent intensity of each well was monitored in the plate reader with measurements taken every 10 min, with 10 s of orbital shaking (amplitude 3) every minute. Cells were grown in the plate for 2 h before being diluted 1:100 into PBS with 0.4% paraformaldehyde. Fixed cells were run on a Novocyte Flow Cytometer (ACEA Biosciences). At least nineteen thousand events were collected for each sample, and data from each sample was analyzed using FCS Express version 4.0 (*De Novo* Software, Los Angeles, CA, United States).

#### F Characterization of biochemical circuits and relative growth

All growth results were generated using a plate reader (Tecan M1000pro). Cells were grown overnight at 37°C at 120° tilt from horizontal with 200 rpm shaking in LB with antibiotics from a single colony, then diluted to a constant OD600 value of 0.5. All cell lines were incubated on ice for 30 min to ensure uniform growth between different cell lines. Cell cultures standardized in this way were further diluted 1:100 into LB media with the appropriate antibiotics and the appropriate density of low-melting-temperature agarose as indicated in the data. The non-resistant cell lines, DH5α and DH5αpro, were grown in media without antibiotics, both constitutive lines and the inducible lines were grown with 30 µg/ml kanamycin. These cells were incubated for 1.5 h at 37°C at 120° tilt from horizontal with 200 rpm shaking until they reached the beginning of exponential growth, at which point they were distributed into a 96-well plate, covered with a lid, and put into the plate reader at 37°C. Total well volume was 200°µl. OD600 and GFP fluorescent intensity of each well was monitored in the plate reader with measurements taken every 10 min. Without shaking, the plate was left undisturbed between measurements. Under intermediate shaking conditions, the plate experienced 10 s of orbital shaking (amplitude 3) every minute. Under high shaking conditions, the plate experienced 50 s of orbital shaking (amplitude 3) every minute. Cells were allowed to grow for 2 h before 50 µl of culture per well was transferred into a new 96-well plate with 150 µl of media already in the plate along with indicated concentrations of tetracycline.

To quantify relative growth, the final density of TetX-expressing cells after 2 h of growth in the second plate was normalized by initial density and the growth of naïve cells cultured under the same conditions Eq. 1.

$$Relative\;Growth=\frac{\frac{OD600_{TetX\;Final}}{OD600_{TetX\;Initial}}}{\frac{OD600_{Naïve\;Final}}{OD600_{Naïve\;Initial}}}$$ (1)

Unless otherwise noted, all results were taken from cells after 120 min of growth in the second plate. Unless otherwise noted, all growth results were replicated 6 times: three sets of duplicated samples were collected on distinct days. No blinding or randomization was performed. A sample size of six was selected according to standard synthetic biology practices of sample replication inside a plate reader for significance testing, but these tests have no established effect size.

#### G Significance testing on relative growth curves

Since we are trying to determine the significance of differences between many points in a relative growth curve, we chose to use a multiple comparisons test instead of a normal *t*-test. For experimental data, each relative growth curve (plotted with respect to noise intensity) was subjected to a Bonferroni multiple comparisons test, which performs significance testing between each pairwise combination of gel concentrations within a relative growth curve, correcting for false positives and producing confidence intervals by adjusting the *p*-value of the test by dividing it by the number of comparisons being made. With this technique, we established confidence intervals between the values in each relative growth curve using a base *p*-value of 0.05. If the curve experienced a significant increase in cell growth followed by a significant decrease, that curve was classified as biphasic.

#### H Measurement of stochastic switching expression in noisy environments

In brief, we followed the distribution of cells that started unimodally “on” and “off”, then used flow cytometry to characterize the bimodal populations of cells that were present at the end of the experiment and counted the proportion of the final population that had switched states. This experiment was broken into two phases to account for the competitive advantage of “on” cells under tetracycline treatment. The first phase starts at a high density of constitutively active cells before measuring their fluorescent gene expression while growing in 0.5% low-melting-temperature agarose. The second phase starts with a low density of cells sorted to be genetically inactive before measuring their fluorescent gene expression while growing in 0.5% low-melting-temperature agarose.

To measure the switching of cells from an “on” state to an “off” state, DH5α cells containing pZ (P_Const_-TetX-GFP) were grown overnight at 37°C at 120°tilt from horizontal with 200 rpm shaking in LB with 30 µg/ml kanamycin from a single colony, then diluted to a constant OD600 value of 0.5. Cells were incubated on ice for 30 min, then further diluted 1:100 into LB media with 30 µg/ml kanamycin and 0.5% low-melting-temperature agarose as indicated in the data. Total well volume was 200 µl. These cells were incubated for 1.5 h at 37°C at 120°tilt from horizontal with 200 rpm shaking until they reached the beginning of exponential growth, at which point they were distributed into a 96-well plate, covered with a lid, and put into the plate reader with 0.625 µg/ml tetracycline at 37°C. OD600 and GFP fluorescent intensity of each well was monitored in the plate reader with measurements taken every 10 min, with 10 s of orbital shaking (amplitude 3) every minute. Cells were grown in the plate for 1 h before being diluted 1:100 into PBS. Some of the cells were analyzed using a Novocyte Flow Cytometer (ACEA Biosciences), and some were used for cell sorting for the second phase of the experiment.

To measure the switching of cells from an “off” state to an “on” state, cells were sorted in an Astrios Cell Sorter (Beckman Coulter), defining the “off” population with a combination of gates on FITC height and width, thresholding reads on side scatter. Once sorted, 2,500 of the “off” cells were put in LB media with 30 µg/ml kanamycin, 0.625 µg/ml tetracycline, and 0.5% low-melting-temperature agarose for a total well volume of 200 µl. The plate was covered with a plastic lid and grown for ∼12 h at 37°C. OD600 and GFP fluorescent intensity of each well was monitored in the plate reader with measurements taken every 10 min, with 10 s of orbital shaking (amplitude 3) every minute. Once the cells had reached the same cell density that they had achieved in the first hour of growth, pZ (P_Const_-TetX-GFP)-containing cells were diluted 1:100 in PBS and analyzed using a Novocyte Flow Cytometer (ACEA Biosciences).

Data from each sample was analyzed using FCS Express version 4.0 (*De Novo* Software, Los Angeles, CA, United States). Results were gated by only including counts that had a linear correlation between area and height measurement on the FITC fluorescent channel. The “on” population and the “off” population were separately determined to separate the two modes seen in the data, and these boundaries were uniformly applied to each replicate. For the “off” to “on” measurement, between five thousand and eighteen thousand counts were collected after gating. For the “on” to “off” measurement, between seventeen thousand and thirty-seven thousand counts were collected after gating.

#### I Simulation of stochastic resonance using a stochastic differential equation

Simulation was performed in MATLAB. Equations were entered using the structures represented in Supplementary Equations S1 or S2. The stochastic differential equation was implemented using the Euler-Maruyama method. 1,000 simulations were done for each condition and averaged together. Average cell growth of each curve with respect to increasing noise was normalized to average cell growth at the lowest noise intensity.

## Data Availability

The original contributions presented in the study are included in the article/Supplementary Material, further inquiries can be directed to the corresponding author.
