# Supplementary material for: Frequency dependent growth of bacteria in living materials
Source: Front Bioeng Biotechnol. 2022 Sep 8;10:948483. doi: 10.3389/fbioe.2022.948483 (PMC9493075; doi:10.3389/fbioe.2022.948483)
Supplement: Supplementary file 1 [file DataSheet1.pdf]

Frequency dependent growth of bacteria in living materials

Daniel D. Lewis[1,2,*], Ting Gong[1,*], Yuanwei Xu[3], Cheemeng Tan[1,+]

[1] Department of Biomedical Engineering, University of California Davis, Davis, CA

[2] Integrative Genetics and Genomics, University of California Davis, Davis, CA

[3] Department of Biomedical Engineering, Peking University, Beijing, China

[+]Corresponding author (cmtan@ucdavis.edu)

*Equal contribution

Supplementary Text

To create a conceptual model for our results, we modified a classical stochastic differential equation (Eq. S1) [1] to describe the effect of stochastic resonance on bacterial growth (Methods- Section I). We opted for a qualitative model due to the lack of mechanistic information about our system.

$$\frac{d\mu}{dt} = \alpha\left(\mu - \sqrt{\frac{\alpha}{\beta}} - \eta\right) - \beta\left(\mu - \sqrt{\frac{\alpha}{\beta}} - \eta\right)^3 + \Lambda\mu\cos(\Omega t) + \sigma\xi(t) \qquad \text{(Eq. S1)}$$

Where μ represents the growth rate (dimensionless), t represents time (dimensionless), α, β, and η are dimensionless parameters that control the bistable state of the system, Λ is a dimensionless parameter that controls the amplitude of a weak oscillatory signal, Ω is the period of a weak oscillatory signal (dimensionless), and σ controls the noise magnitude (dimensionless).

The first two terms on the right-hand side of Eq. S1 represent bistable growth caused by the feedback between importation, degradation, and translation-inhibition activity of antibiotics as described by Deris et. al. [2]. To verify the bistability of the equation, we plotted the output of the positive and negative function terms to identify the stable and unstable fixed points (**Supp. Fig. 11A**). We also implemented a simpler equation (Eq. S2) to represent non-bistable (i.e. monostable) growth of bacteria in the absence of tetracycline.

$$\frac{d\mu}{dt} = \alpha^* - \beta^*\mu + \Lambda\mu\cos(\Omega t) + \sigma\xi(t) \qquad \text{(Eq. S2)}$$

Where α^* and β^* are dimensionless parameters. A parameter set for Eq. S2 was empirically derived to quantitatively match the integral of the growth rates from Eq. S1. The stability diagram of Eq. S2 was plotted under the chosen parameters, demonstrating that it is not bistable (**Supp. Fig. 11B**).

Next, Eq. S1 and S2 were used to simulate bacterial growth with weak periodic perturbation of growth rates. Hydrogel encapsulation was modeled as increasing the noise in bacterial growth by causing noisy TetX expression[3]. Simulations showed that at a narrow range of amplitude and frequency, the relative bacterial growth rate over a range of simulated gel densities was enhanced (**Supp. Fig. 11C**). Simulated bacterial

growth without bistability did not experience frequency and noise specific enhancements (**Supp. Fig. 11D**).

The simulated growth of bacteria experiencing frequency-dependent enhancement was plotted against the range of simulated gel densities (**Supp. Fig. 11E** black line). Under conditions where relative growth enhancement was reported, simulated growth was found to be biphasic as simulated hydrogel densities increased (**Supp. Fig. 11E** black line). Removing the weak periodic force (**Supp. Fig. 11E** dark grey), changing the underlying bistability (**Supp. Fig. 11E** mid grey), or altering the periodic forcing frequency (**Supp. Fig. 11E** light grey) eliminated the biphasic growth with respect to simulated hydrogel density. These simulations demonstrate that stochastic resonance may explain the biphasic, frequency-dependent bacterial growth curves that we experimentally observed in tetracycline-resistant bacteria.

Supplementary Figure Legends

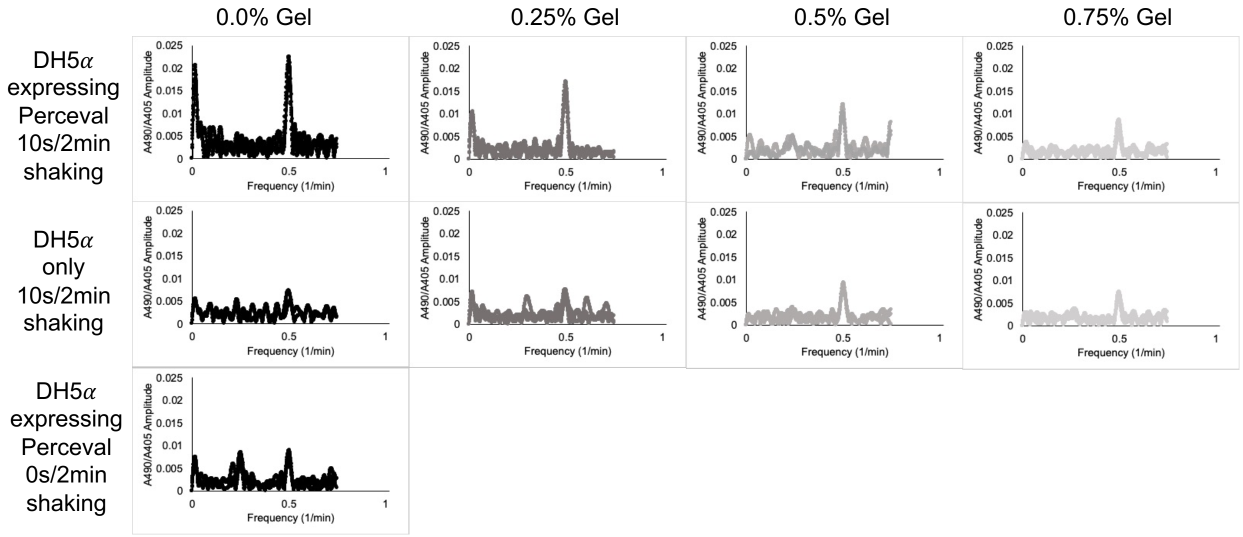

Supplementary Figure 1. Frequency spectra of cells for A490/A405 ratio

Graphs representing Fourier transformations of the oscillating ratio of absorbance at 490nm and absorbance at 405nm. The ratio of A490/A405 has been shown to represent the ratio of ATP to ADP in cells expressing Perceval. These results show that the frequency spectra of cells with Perceval and cells without Perceval can be distinguished at 0.0% and 0.25% gel, but not 0.5% or 0.75% gel. Furthermore, the frequency spectra of cells expressing Perceval and shaken at 10s/min can be distinguished from cells expressing Perceval and shaken at 0s/min. All graphs show 3 biological replicates, except for DH5α cells in 0.0% gel with 10s/min shaking, which shows 2 biological replicates.

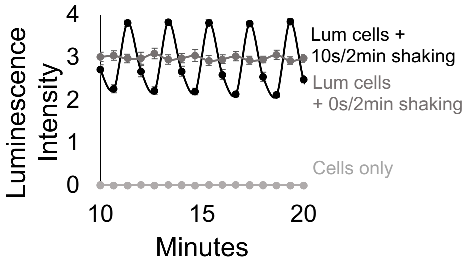

Supplementary Figure 2. Bacteria expressing a luminescent reporter under periodic and heterogeneous conditions.

Metabolic activity of cells in media with .5% low-melting-temperature agarose as measured by cells expressing luciferase, which uses an ATP- and oxygen-dependent reaction to catalyze the production of light. Periodic shaking induces an approximately two-fold periodic change in luminescence (Methods- section D). Along with fluctuations in Perceval A490/A405 levels, these results suggest that periodic shaking of cells imparts periodic perturbation of metabolism in those cells. Points represent mean values, and error bars represent standard error of the mean. Mean and SEM are calculated using six biological replicates.

(A)

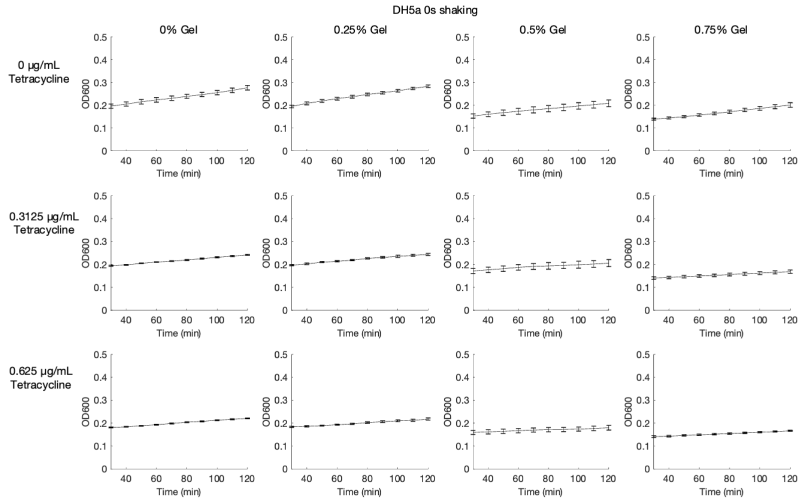
DH5a 0s shaking
0% Gel
0.25% Gel
0.5% Gel
0.75% Gel
0 µg/mL Tetracycline
0.3125 µg/mL Tetracycline
0.625 µg/mL Tetracycline
OD600
Time (min)

(B)

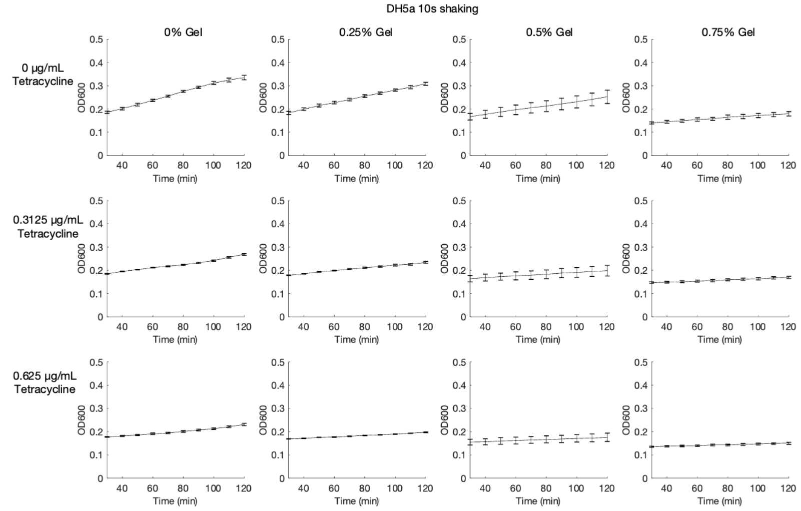
DH5a 10s shaking
0% Gel
0.25% Gel
0.5% Gel
0.75% Gel
0 µg/mL Tetracycline
0.3125 µg/mL Tetracycline
0.625 µg/mL Tetracycline
OD600
Time (min)

(C)

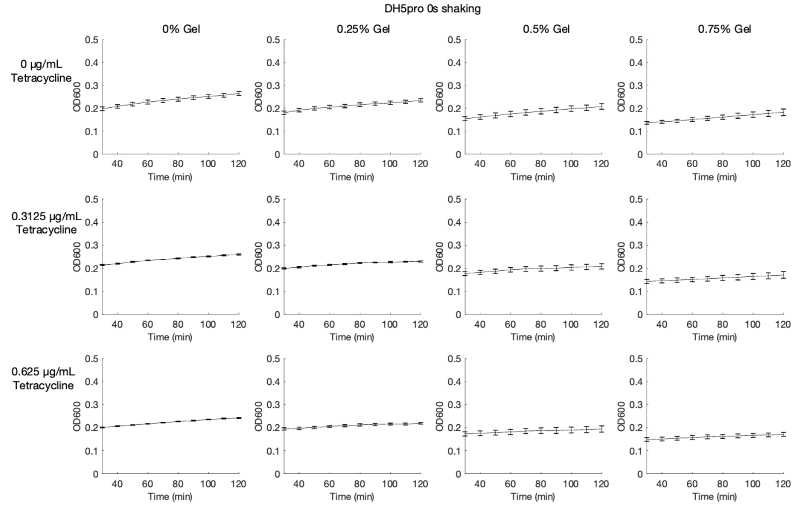
DH5pro 0s shaking
0% Gel
0.25% Gel
0.5% Gel
0.75% Gel
0 µg/mL Tetracycline
0.3125 µg/mL Tetracycline
0.625 µg/mL Tetracycline
OD600
Time (min)

(D)

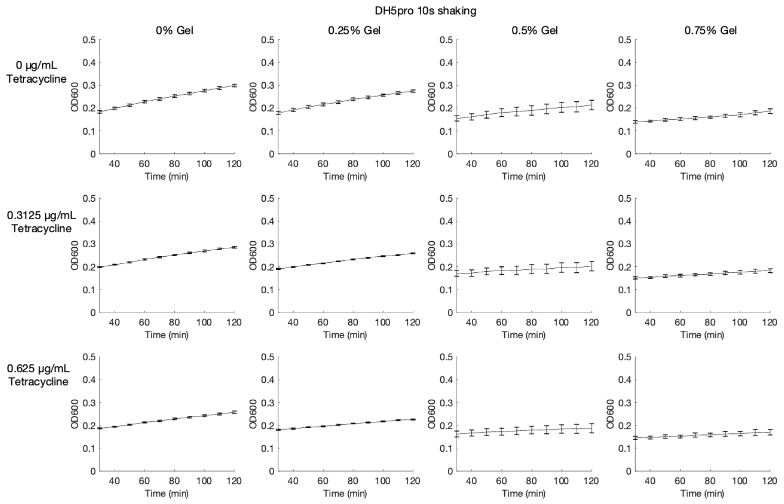

Supplementary Figure 3. Changes in OD600 over time for DH5α and DH5αpro cells grown in hydrogel and tetracycline.

(A) Changes in OD600 over time for DH5α grown without shaking.

(B) Changes in OD600 over time for DH5α grown with 10 seconds shaking per minute.

(C) Changes in OD600 over time for DH5αpro grown without shaking.

(D) Changes in OD600 over time for DH5αpro grown with 10 seconds shaking per minute.

(A-D) Cell grown in LB with 0, 0.25, 0.5, or 0.75% gel along with 0 µg/mL, 0.3125 µg/mL, or 0.625 µg/mL tetracycline. Points represent mean values, and error bars represent standard error of the mean. Mean and SEM are calculated using six biological replicates.

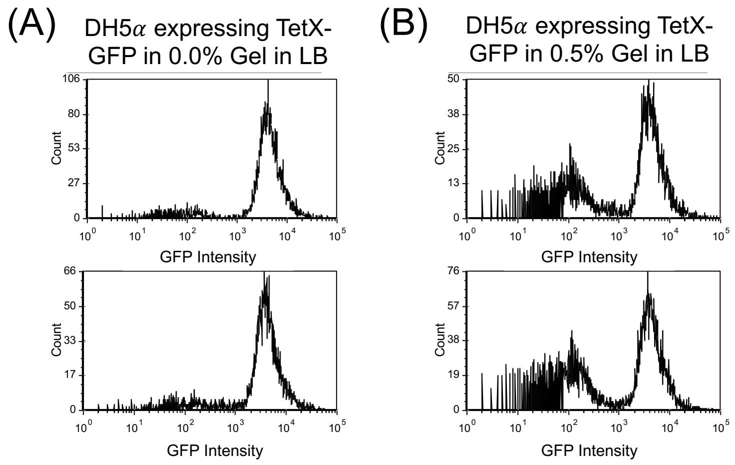

Supplementary Figure 4. Hydrogel encapsulation causes heterogeneous protein expression.

(A) Histograms of GFP intensity of DH5α cells constitutively expressing TetX-GFP in LB with 0% gel. All biological replicates are shown.

(B) Histograms of GFP intensity of DH5α cells constitutively expressing TetX-GFP in LB with 0.5% gel. All biological replicates are shown.

(A&B) Histograms represent 5000-8000 events after filtering.

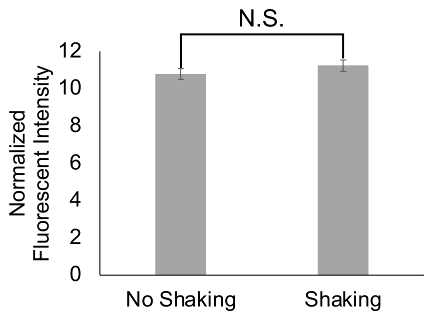

Supplementary Figure 5: Periodic perturbation does not increase gene module expression.

Fluorescent intensity of TetX-GFP normalized by OD600 of cells after 1 hour of growth. Two-tailed, equal variance t-test returns *p*-value = 0.345 N.S. stands for not significant. Bar height represents mean values, and error bars represent standard error of the mean. Mean and SEM are calculated using eight biological replicates.

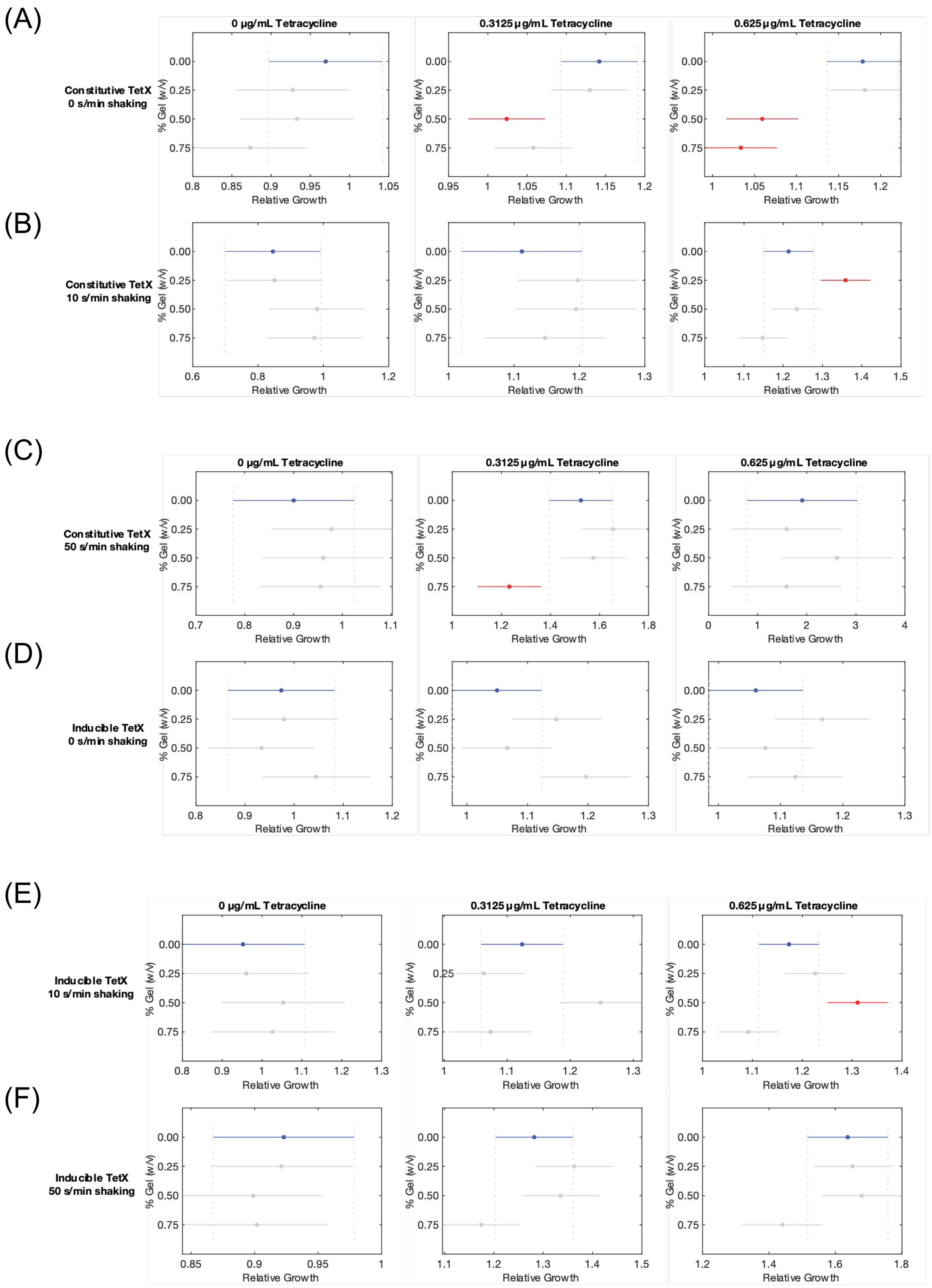

Supplementary Figure 6. Significance testing of relative growth curves.

(A) Confidence intervals of relative growth for bacteria constitutively expressing stable TetX grown with 0 s/min shaking (left panel) 0 µg/mL tetracycline (middle panel) 0.3125 µg/mL tetracycline (right panel) 0.625 µg/mL tetracycline

(B) Confidence intervals of relative growth for bacteria constitutively expressing stable TetX grown with 10 s/min shaking (left panel) 0 µg/mL tetracycline (middle panel) 0.3125 µg/mL tetracycline (right panel) 0.625 µg/mL tetracycline

(C) Confidence intervals of relative growth for bacteria constitutively expressing stable TetX grown with 50 s/min shaking (left panel) 0 µg/mL tetracycline (middle panel) 0.3125 µg/mL tetracycline (right panel) 0.625 µg/mL tetracycline

(D) Confidence intervals of relative growth for bacteria inducibly expressing stable TetX grown with 0 s/min shaking (left panel) 0 µg/mL tetracycline (middle panel) 0.3125 µg/mL tetracycline (right panel) 0.625 µg/mL tetracycline

(E) Confidence intervals of relative growth for bacteria inducibly expressing stable TetX grown with 10 s/min shaking (left panel) 0 µg/mL tetracycline (middle panel) 0.3125 µg/mL tetracycline (right panel) 0.625 µg/mL tetracycline

(F) Confidence intervals of relative growth for bacteria inducibly expressing stable TetX grown with 50 s/min shaking (left panel) 0 µg/mL tetracycline (middle panel) 0.3125 µg/mL tetracycline (right panel) 0.625 µg/mL tetracycline

(A-F) Significance intervals calculated by Bonferroni multiple comparisons test within the growth curve (Methods- Section G).

(A)

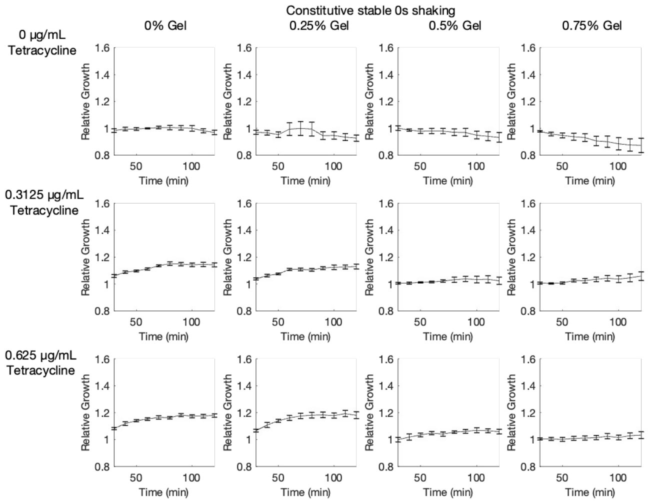

Constitutive stable 0s shaking
0% Gel
0.25% Gel
0.5% Gel
0.75% Gel
0 μg/mL Tetracycline
0.3125 μg/mL Tetracycline
0.625 μg/mL Tetracycline
Relative Growth
Time (min)

(B)

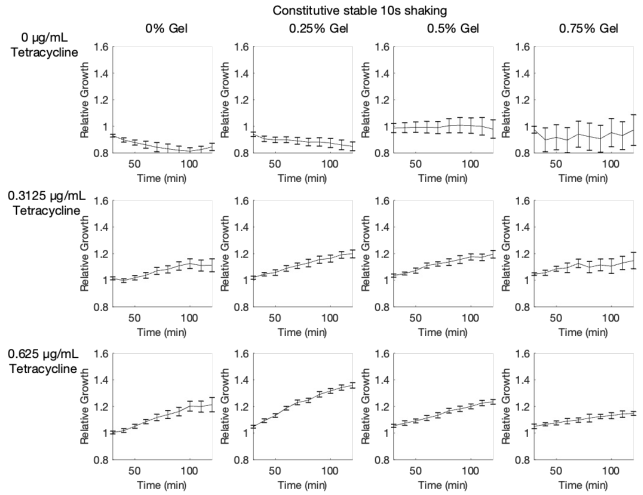

Constitutive stable 10s shaking
0% Gel
0.25% Gel
0.5% Gel
0.75% Gel
0 μg/mL Tetracycline
0.3125 μg/mL Tetracycline
0.625 μg/mL Tetracycline
Relative Growth
Time (min)

(C)

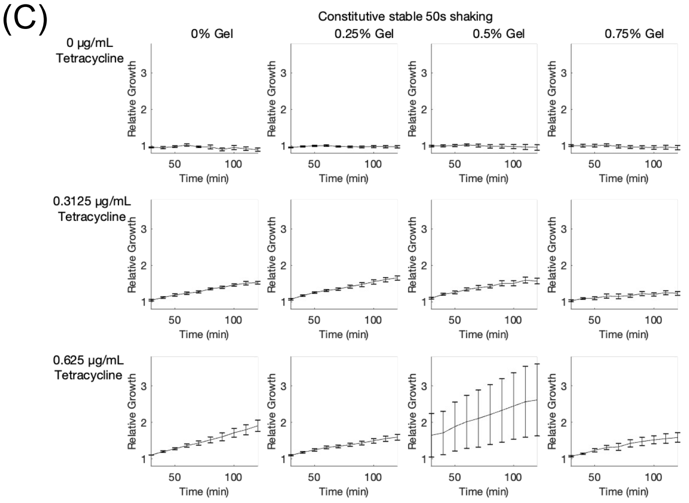

Constitutive stable 50s shaking
0% Gel
0.25% Gel
0.5% Gel
0.75% Gel
0 μg/mL Tetracycline
0.3125 μg/mL Tetracycline
0.625 μg/mL Tetracycline
Relative Growth
Time (min)

(D)

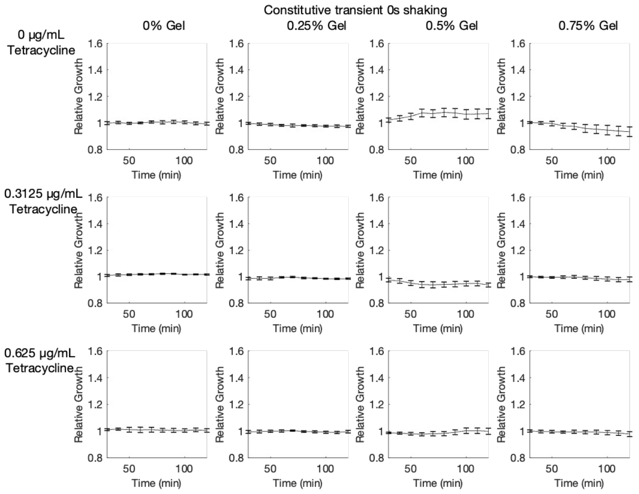

(E)

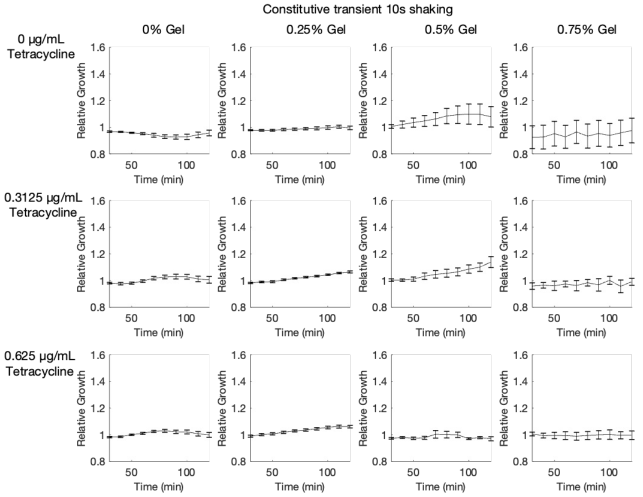

Supplementary Figure 7. Changes in relative growth over time for DH5α cells expressing TetX grown in hydrogel and tetracycline.

(A) Changes in relative growth over time for DH5α cells expressing TetX constitutively, grown without shaking.

(B) Changes in relative growth over time for DH5α cells expressing TetX constitutively, grown with 10s/min shaking. Large reading at an early time point of 0.75% gel and 0 μg/mL tetracycline correspond to a bubble interfering with reading.

(C) Changes in relative growth over time for DH5α cells expressing TetX constitutively, grown with 50s/min shaking.

(D) Changes in relative growth over time for DH5α cells expressing TetX-ssra constitutively, grown without shaking. Degradation tag (ssra) makes TetX unstable.

(E) Changes in relative growth over time for DH5α cells expressing TetX-ssra constitutively, grown with 10s/min shaking. Degradation tag (ssra) makes TetX

unstable.

(A-E) Cell grown in LB with 0, 0.25, 0.5, or 0.75% gel along with 0 μg/mL, 0.3125 μg/mL, or 0.625 μg/mL tetracycline. Points represent mean values, and error bars represent standard error of the mean. Mean and SEM are calculated using six biological replicates except for (C), which is calculated using four biological replicates.

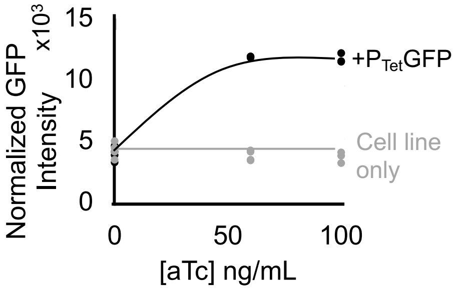

Supplementary Figure 8. Inducible circuit characterization.

Characterization of P_{Tet} producing GFP in DH5αpro cells in response to anhydrous tetracycline. Cell line only control is background fluorescence of DH5αpro cells.

Points represent individual replicates, line highlights trend. There are four replicates per condition.

(A)

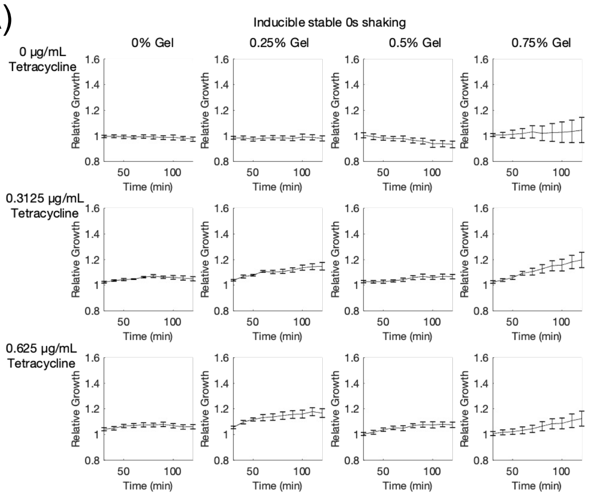
Inducible stable 0s shaking
0% Gel
0.25% Gel
0.5% Gel
0.75% Gel
0 µg/mL Tetracycline
0.3125 µg/mL Tetracycline
0.625 µg/mL Tetracycline
Relative Growth
Time (min)

(B)

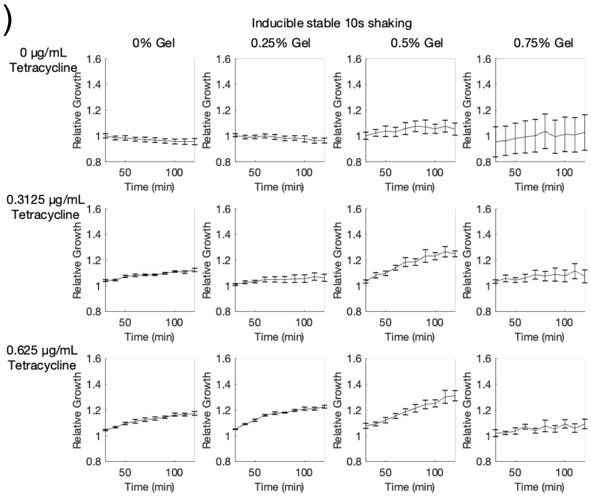
Inducible stable 10s shaking
0% Gel
0.25% Gel
0.5% Gel
0.75% Gel
0 µg/mL Tetracycline
0.3125 µg/mL Tetracycline
0.625 µg/mL Tetracycline
Relative Growth
Time (min)

(C)

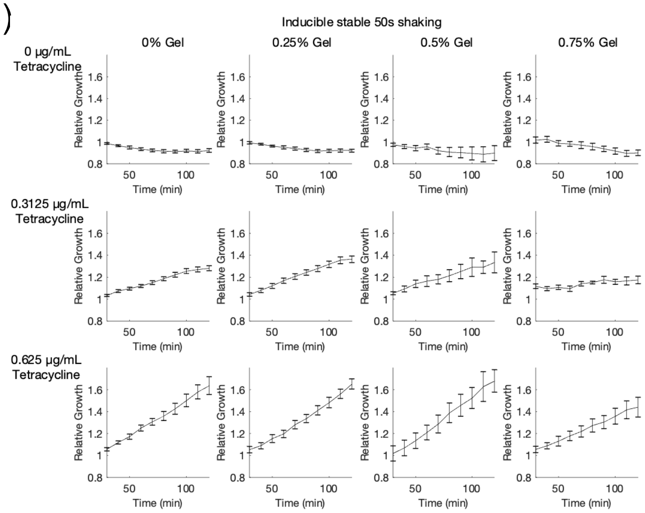
Inducible stable 50s shaking
0% Gel
0.25% Gel
0.5% Gel
0.75% Gel
0 µg/mL Tetracycline
0.3125 µg/mL Tetracycline
0.625 µg/mL Tetracycline
Relative Growth
Time (min)

(D)

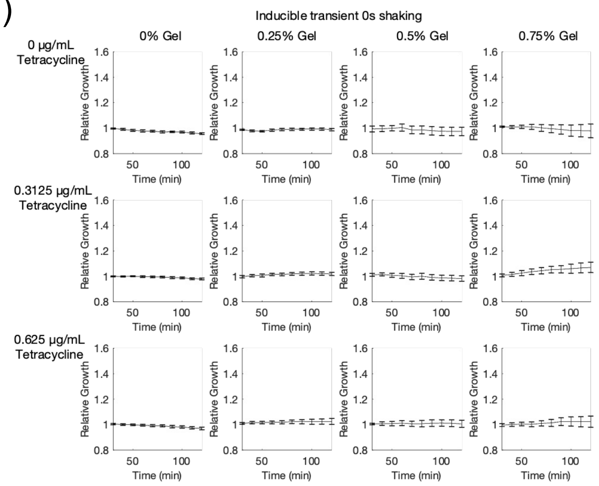

(E)

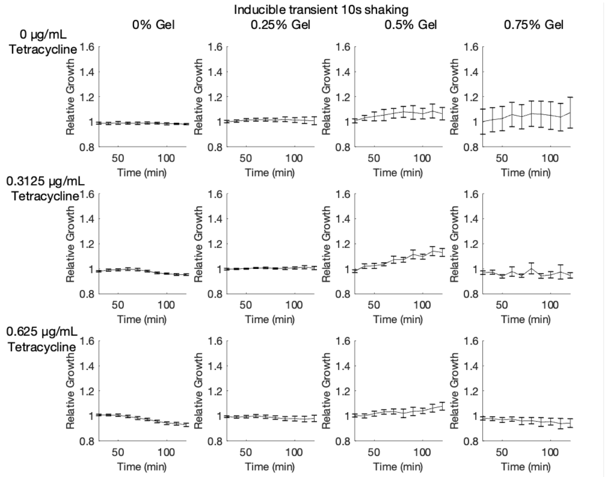

Supplementary Figure 9. Changes in OD600 over time for DH5αpro cells expressing TetX grown in hydrogel and tetracycline.

(A) Changes in relative growth over time for DH5αpro cells expressing TetX upon tetracycline induction, grown without shaking.

(B) Changes in relative growth over time for DH5αpro cells expressing TetX upon tetracycline induction, grown with 10s/min shaking.

(C) Changes in relative growth over time for DH5αpro cells expressing TetX constitutively, grown with 50s/min shaking.

(D) Changes in relative growth over time for DH5αpro cells expressing TetX-ssra upon tetracycline induction, grown without shaking. Degradation tag (ssra) makes TetX unstable.

(E) Changes in relative growth over time for DH5αpro cells expressing TetX-ssra upon tetracycline induction, grown with 10s/min shaking. Degradation tag (ssra) makes TetX

unstable.

(A-E) Cell grown in LB with 0, 0.25, 0.5, or 0.75% gel along with 0 μg/mL, 0.3125 μg/mL, or 0.625 μg/mL tetracycline. Points represent mean values, and error bars represent standard error of the mean. Mean and SEM are calculated using six biological replicates.

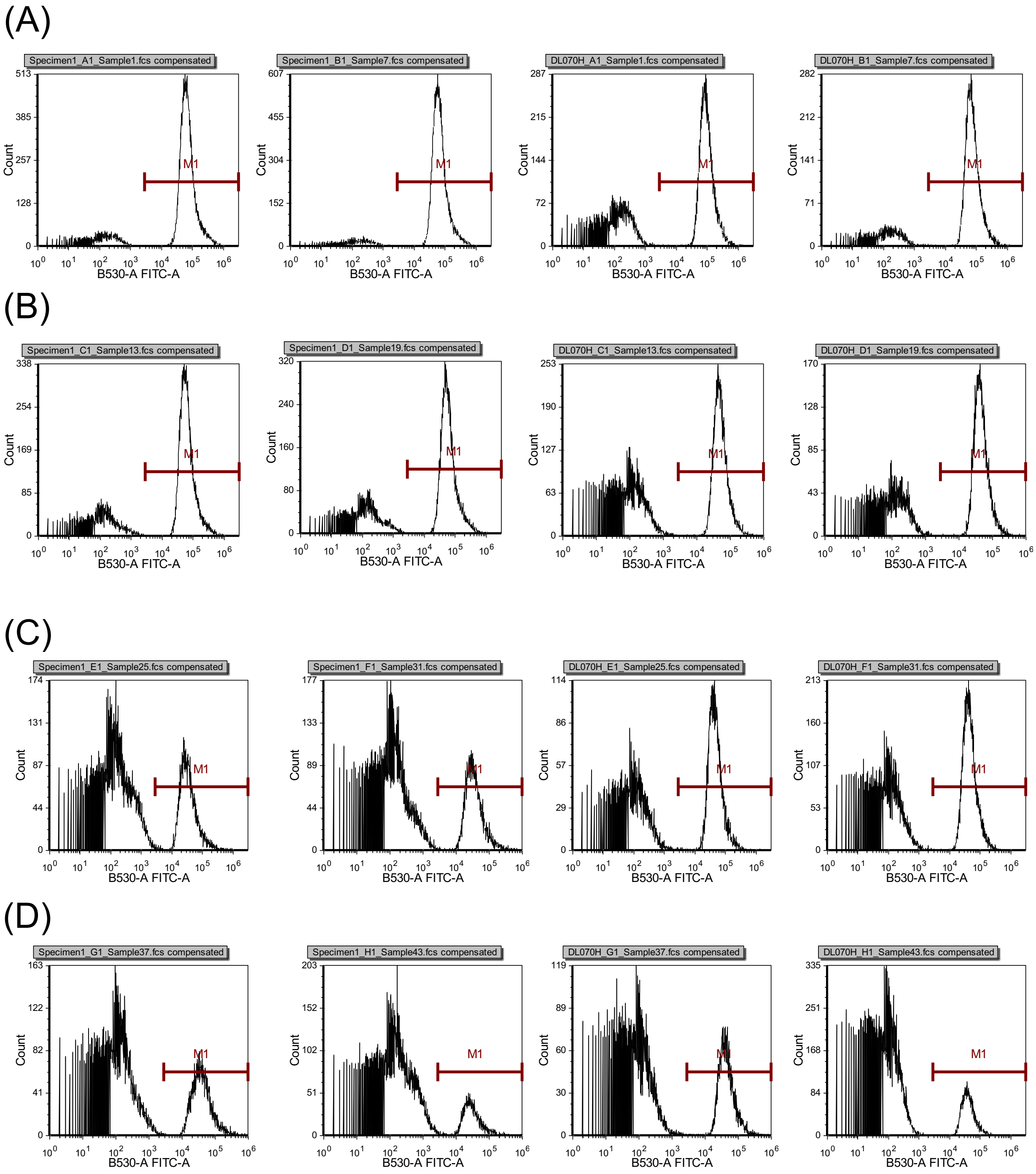

Supplementary Figure 10. Increased hydrogel density causes greater expression heterogeneity

(A) Histograms of GFP intensity of DH5α cells constitutively expressing TetX-GFP in LB with 0% gel. All biological replicates are shown.

(B) Histograms of GFP intensity of DH5α cells constitutively expressing TetX-GFP in LB with 0.25% gel. All biological replicates are shown.

(C) Histograms of GFP intensity of DH5α cells constitutively expressing TetX-GFP in LB with 0.5% gel. All biological replicates are shown.

(D) Histograms of GFP intensity of DH5α cells constitutively expressing TetX-GFP in LB with 0.75% gel. All biological replicates are shown.

(A-D) Histograms represent 5000-20000 events after filtering.

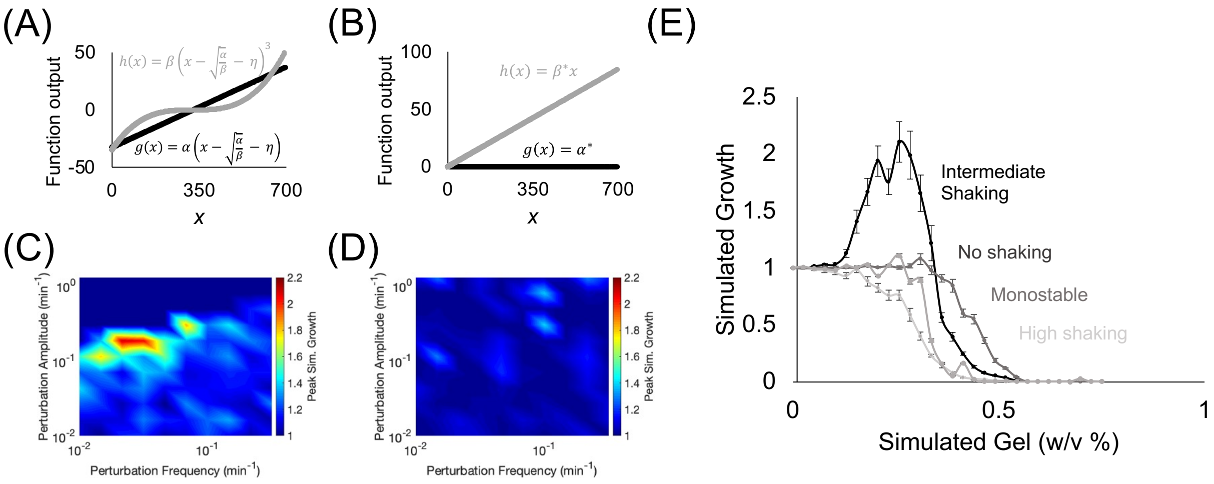

Supplementary Figure 11. A conceptual model may explain the frequency-specific biphasic bacterial growth

(A) Output of the positive (h(x), grey line) and negative function (g(x), black line) that represent a system with two stable fixed points.
(B) Output of the positive (h(x), grey line) and negative function (g(x), black line) that represent a system with one stable fixed point.
(C) Heat plot showing the simulated growth of bacteria at different frequencies and amplitudes of periodic perturbation. Color indicates the maximum growth of bacteria over a range of simulated hydrogel densities normalized by the growth of bacteria at the lowest gel density. Red areas show regions where stochastic resonance is causing biphasic changes in bacterial growth with respect to gel density. Surface plot values represent mean values. The mean is calculated using two hundred simulation replicates.
(D) Heat plot showing the simulated growth of bacteria that cannot achieve bistable growth at different frequencies and amplitudes of periodic perturbation. Color indicates the maximum growth of bacteria over a range of simulated hydrogel densities normalized by the growth of bacteria at the lowest gel density. The results indicate that stochastic resonance does not occur under these conditions. Surface plot values represent mean values. The mean is calculated using two hundred simulation replicates.
(E) Simulated bacterial growth in different hydrogel densities. Black line represents simulation with the amplitude, frequency, and bistable growth required for stochastic resonance. Black line shows growth under stochastic resonance conditions causes a biphasic relationship between the total growth in the system and the simulated gel density, similar to the 10 s/min shaking conditions in Fig. 3. Dark grey represents simulation with a periodic amplitude of zero, similar to the 0 s/min shaking conditions. Intermediate grey represents a simulation with a monostable system. Light grey represents a simulation with a reduction in the frequency of periodic perturbation. Results demonstrate that changing periodic forcing amplitude, frequency, or the underlying bistability of growth eliminates biphasic growth with respect to simulated gel density. Points represent mean values, and error bars represent standard error of the mean. Mean and SEM are calculated using two hundred simulation replicates.

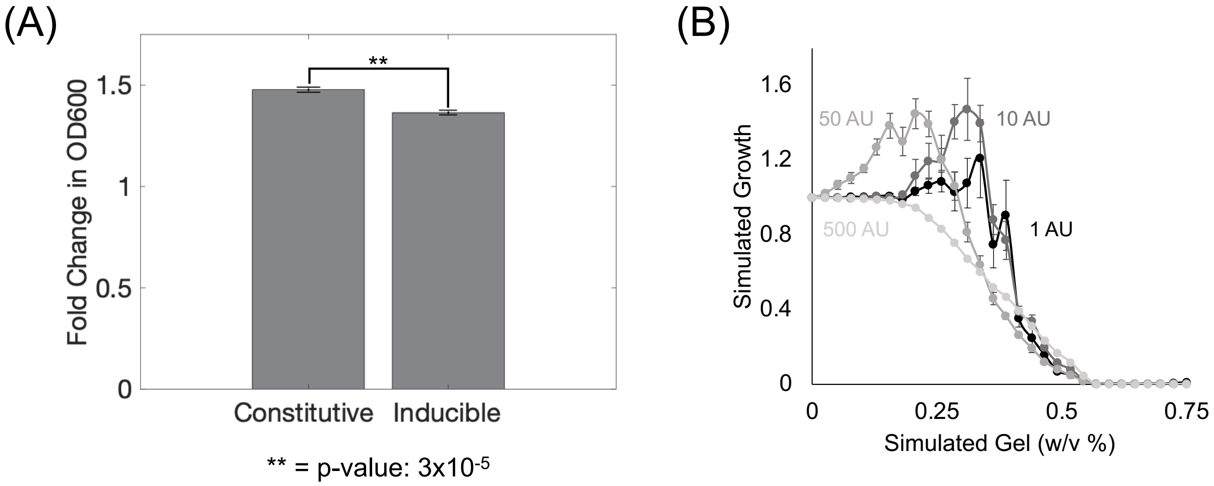

Supplementary Figure 12. The change in basal growth rate associated with inducible TetX production is sufficient to shift biphasic relative growth curve

(A) OD600 values of constitutive and inducible TetX producing cells normalized by initial OD600. Comparison was performed at 0.625 μg/mL tetracycline, 0% gel, and 0 s/min shaking to characterize the difference in basal growth rate between the two lines under tetracycline treatment. A single-tailed t-test shows that the constitutive TetX producers have a significantly higher growth rate than the inducible TetX producers (p-value: 3×10^{-5}). Points represent mean values, and error bars represent standard error of the mean. Mean and SEM are calculated using six biological replicates.

(B) Simulation of stochastic resonance governing bacterial growth with different basal growth rates. Basal growth rate is controlled by changing the value of η in Eq. S1. Lines are labeled with the value of η used in the simulation. Results show that decreasing the basal growth rate of a bacterial strain affected by stochastic resonance is sufficient to increase the optimal gel density for bacterial growth. These results suggest that the change in basal growth rate shown in Supp. Fig. 12A is responsible for the shifted biphasic relative growth curve seen in Fig. 3G (red lines). AU = arbitrary units.

Points represent mean values, and error bars represent standard error of the mean. Mean and SEM are calculated using two hundred simulation replicates.

References

(1) Gammaitoni, L.; Hanggi, P.; Jung, P.; Marchesoni, F. Stochastic resonance. *Rev Mod Phys* **1998**, *70* (1), 223-287. DOI: DOI 10.1103/RevModPhys.70.223.
(2) Deris, J. B.; Kim, M.; Zhang, Z.; Okano, H.; Hermsen, R.; Groisman, A.; Hwa, T. The innate growth bistability and fitness landscapes of antibiotic-resistant bacteria. *Science* **2013**, *342* (6162), 1237435. DOI: 10.1126/science.1237435.
(3) Kiviet, D. J.; Nghe, P.; Walker, N.; Boulineau, S.; Sunderlikova, V.; Tans, S. J. Stochasticity of metabolism and growth at the single-cell level. *Nature* **2014**, *514* (7522), 376-379. DOI: 10.1038/nature13582 From NLM Medline.
